# Coastal Bathymetric Sounding in Very Shallow Water Using USV: Study of Public Beach in Gdynia, Poland

**DOI:** 10.3390/s23094215

**Published:** 2023-04-23

**Authors:** Artur Makar

**Affiliations:** Department of Navigation and Hydrography, Polish Naval Academy, 81-127 Gdynia, Poland; artur.makar@amw.gdynia.pl

**Keywords:** hydrographic surveys, bathymetry, survey planning, Electronic Navigational Chart, Digital Sea Bottom Model

## Abstract

The bathymetric surveys executed with a use of small survey vessels in limited water areas, including offshore areas, require precise determination of the geospatial coordinates of the seabed which is a synthesis of, among others, determining the position coordinates and measuring the depth. Inclination of the seabed and the declining depth make manoeuvring of the sounding vessel, e.g., a hydrographic motorboat or Unmanned Survey Vehicle (USV), in shallow water impossible. Therefore, it is important to determine the minimal depth for the survey resulting from the draught of the sounding vessel and the limits of the sounding area. The boundaries also result from the dimensions of the sounding vessel, its manoeuvring parameters and local water level. Type of the echosounder used in the bathymetric survey is a decisive factor for the sounding profile planning and the distances between them and the survey vessel for the possibility performing the measurements in shallow water. Electronic Navigational Chart (ENC) was used to determine the water area’s boundaries, and the safety contours were determined on the basis of the built Digital Sea Bottom Model (DSBM). The safety contour, together with the vessel’s dimensions, its manoeuvring parameters and the hydrometeorological conditions, limit the offshore area in which the measurement can be performed. A method of determining boundaries of the survey performed by a USV equipped with SingleBeam EchoSounder (SBES) on survey lines perpendicular to the coastal line are presented in the paper in order to cover the water area with the highest amount of measurement data, with the USV’s navigational safety taken into consideration. The measurements executed on the municipal beach served verification of the DSBM.

## 1. Introduction

Small surface vessels increasingly often replace cutters and hydrographic motorboats in bathymetric surveys in the inshore zone [1,2,3,4,5,6,7,8,9,10]. Small size and high manoeuverability, as well as one-man personnel, are among their main advantages. They are often fitted out with SBES for depth measurement owing to the high precision positioning and the vessel guiding along the survey lines (line keeping) [11,12]. This is possible owing to applying the geodetic positioning systems [13,14] in an open upper hemisphere, which enables determining the mobile position coordinates [15].

The International Hydrographic Organization (IHO) [16,17] and local [18,19] requirements specify that 100% of the seafloor must be covered with measurements, which can be understood as a measurement with a Side Scan Sonar (SSS) or an MultiBeam Echosounder (MBES), although the device records a cloud of discrete points. The distance between points of acoustic wave reflection from the seafloor is much smaller than for SBES, and it depends on the survey vessel velocity, the sound beam arrangement and the depth. When measurements are made with an SBES perpendicularly to the vessel’s direction of movement, the distance between the soundwave reflection points depends on the distance between sounding lines and ranges from a few to about a dozen metres. Although the requirement of 100% coverage with data is not fulfilled, measurements with USV equipped with SBES are popular and provide an alternative to MBES mounted on larger autonomous vessels.

A general methodology of planning survey lines [20,21] and good practice in hydrography [22] focus on the position of profiles and other objects (wharf, breakwater) in relation to each other and to the coastline. The maximum distance between profiles is determined, as are the rules of their concentration when an object is detected on the seafloor or in the case of shallowing. Manual steering of a hydrographic motorboat (and doing it perpendicularly to a wharf or the coastline) is an onerous task to the steersman. Therefore, survey profiles are planned at the smallest required distance, which is usually several times greater than between the profiles planned for measurements performed with USV.

Registering geospatial data is another stage of bathymetric surveys. It is important to guide a survey vessel along the sounding lines planned at the first measurement stages. As a navigation problem, it consists in maintaining the vessel’s trajectory (its position and the position of its positioning system) on the planned route. The accuracy of keeping along the survey lines is affected by the positioning system accuracy, the helmsman’s skills (both with a motorboat and a USV in a manual mode), a survey vessel’s manoeuvering parameters, hydrometeorological conditions as well as the model of navigation automation and the basin characteristics (wharf, beach).

Planning bathymetric surveys, including survey lines, is easier in an open basin than on a limited one. It is particularly difficult to plan profiles in harbours and marinas because of the basin diversity. There are frequently ships anchored in a harbour basin, which prevent performing a survey of the whole basin. Mooring sites in marinas are made as Y-booms with a small distance between them [23,24,25]. The width of a mooring site is a manoeuverability-limiting factor.

It is important to determine the land-side border when planning measurements in the inshore area, according to the methodology [19] and perpendicularly to the coast. For manual steering, longer profiles can be planned, which include some land. While controlling the depth with an echosounder, a turn is performed to the next survey line to ensure the survey vessel’s safety. Their limit in measurement automatic mode should be established while taking into account the water level, the safety contour [26,27,28] and the manoeuvering parameters [29,30,31,32]. This will enable performing the measurements as close to the coastline as possible.

**Figure 1:**
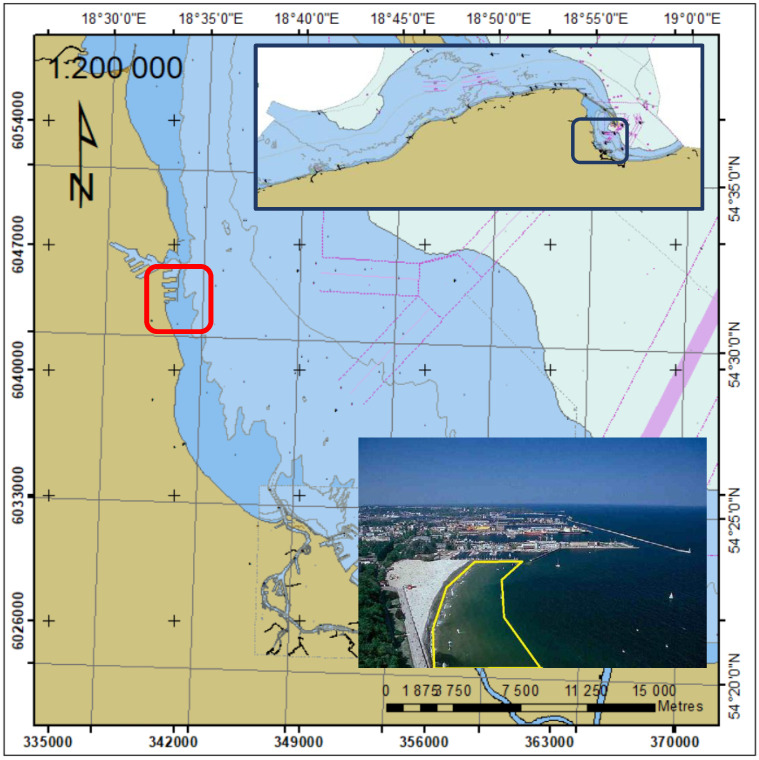
Location of bathymetric surveys in coastal area in Gdynia.

Bathymetric surveys were performed in the basin adjacent to the municipal beach, between the southern breakwater of the marina and the sea boulevard in May 2020. The area of research is shown in Figure 1.

The paper is structured as follows. The introduction to the study, purpose and study area are provided in Section 1. Section 2 details general methodology of survey lines’ planning using SBES, planning of survey lines in restricted area, water level observation and prediction, Digital Sea Bottom Model and the safety contour. Section 3 presents the results of the bathymetric surveys (trajectory of the USV) limited using the following methods: coastline on the basis of the ENC, hydrographic reconnaissance and safety contour on the basis of DSBM. Section 4 concludes the paper.

## 2. Materials and Methods

### 2.1. General Methodology of Survey Lines’ Planning Using SBES

As SBESs are used on small vessels (a motorboat, USV), measurements are performed in inshore areas. These include maritime hydrotechnical structures and inshore areas. Basic profiles in the hydrotechnical structure area are designed in parallel to the hydrotechnical structure line. Control profiles are designed perpendicularly to basic profiles [20,21].

Basic survey lines in inshore basins are usually designed perpendicularly to the seafloor relief, general direction of contour lines (isobaths) or the coastline. Control survey lines are designed perpendicularly to basic lines. Basic survey lines in basins with a varied coastline are designed at an angle of 45° to the general direction of contour lines or the coastline [20,21].

Surveys of shallows involve determining the value and position of the smallest depth, determining the boundaries of shallows and determining the relief and types of their boundaries. To this end, a dense profile network is designed according to the following principles: a basin near the shallow border is covered with a network of mutually perpendicular profiles spaced at twice less than in the basic survey profile network; as the survey work progresses, the directions and distances of profiles are determined accurately [20,21].

A higher concentration of measurement survey lines is used for SBES measurements in order to determine the course of contour lines and forms of seafloor relief with greater precision. To this end, additional lines are created: at sites where signs of shallows are present; in basins with varied depths and seafloor relief; at places where directions of the planned profiles are similar to the courses of survey lines, which prevents their correct plotting; on the axes of canals and fairways [20,21].

### 2.2. Planning of Survey Lines in Restricted Areas

**Figure 2:**
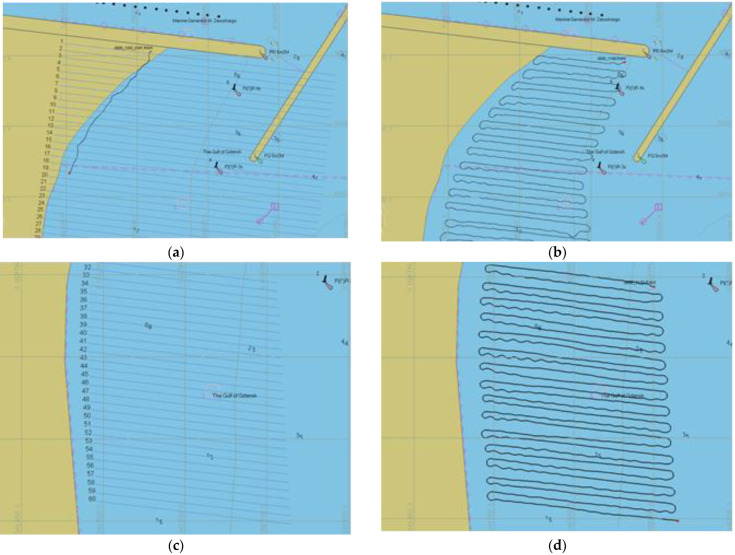

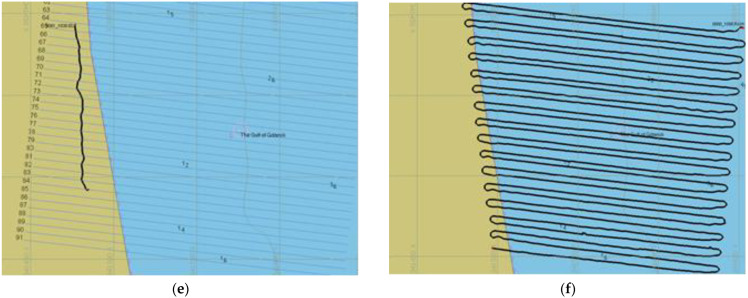
Stages of planning the survey lines in the restricted area: (**a**,**e**) rectangular area including shallow water and land area, (**c**) area limited using the ENC, (**b**,**d**,**f**) trajectory of the USV on reduced survey lines.

Planning of parallel survey lines with a use of typical hydrographic systems QINSy (QPS, Zeist, The Netherlands), HYPACK (HYPACK, Middletown, CT, USA) and EIVA (EIVA a/s, Skanderborg, Denmark) consists of determination of the base line, number of lines and distances between them. In the first stage, it can involve an inaccessible area for the sounding vessel as for instance shallows (shoal, coast), navigational marks or hydrotechnical structures. In manual mode, based on the information about the depth or on observation of the navigational obstacles, the helmsman makes a turn onto the next sounding line. It is possible to limit the sounding area on the basis of ENC or the reconnaissance, performed by a USV. It consists of a USV steering so that it moves safely but as close to the shallows or navigational dangers (stones, rocks, boughs of trees) as possible. The reconnaissance method can be useful in the areas where ENCs have not been developed (inland water areas, mainly lakes) or where the USV’s navigation is highly probable to be in jeopardy. Figure 2 presents stages of planning profiles in three surveyed areas using reconnaissance and ENC methods.

### 2.3. Water Level Observation and Prediction

Considering the dynamic changes of the seawater levels caused by anemobaric (atmospheric pressure, wind direction and velocity), hydrological (river basins) factors, and tidal elements, obtaining information on the current water level in an area where bathymetric measurements are performed is one of the most important tasks of each hydrographer. This information allows for determining the measured depths relative to the reference level for bathymetric surveys, known as the chart datum. Further, during data processing, this information also allows for handling the bathymetric data referred to in the vertical reference system, which is part of the system used in the National Spatial Reference System [33] areas in the part associated with the creation and updating of data contained in maritime cartographic documents and in navigation databases of the Electronic Chart Display and Information System (ECDIS) and Electronic Chart System ECS.

Observations are performed at stream gauge sites in harbours in cooperation with the state maritime administration (harbour authorities) [34,35,36,37]. The water level observation is executed with staff gauges and it is registered with mareographs, which allow for the continuous registration of water level changes. Information on the water level and its other parameters are available on the Internet at various, usually one-hour intervals [38,39,40,41].

The following are used in field applications, where local conditions require information on water level fluctuations:

- mobile water level gauge stations, often referred to as tide-gauge or tide-stations. They usually make use of special pressure and temperature sensors, which measure changes in the water column and use the registered changes to convert the data, referring them to the water level changes;
- the RTK-tide method which enables determination of the water level based on the altitude of a GNSS receiver antenna in a chosen reference system and reducing (converting) it to a local vertical altitude system [41,42,43].

**Figure 3:**
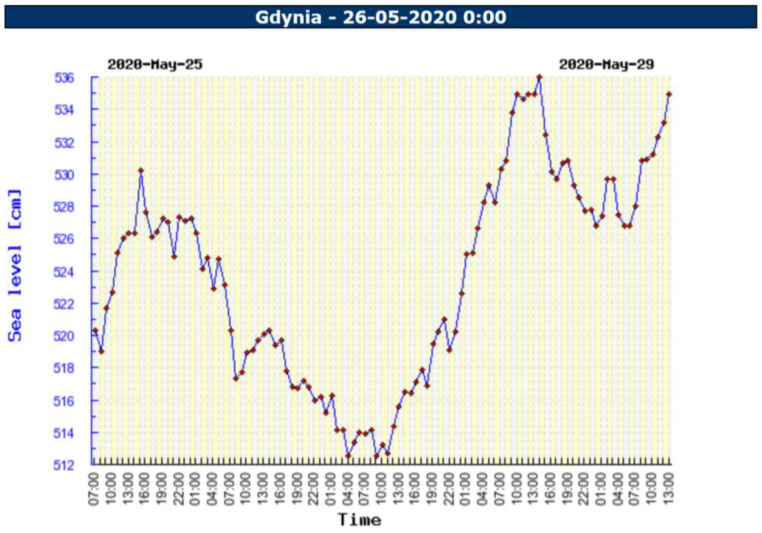
Sea level recorded in the water station in Gdynia [35].

Water level, on the basis of information available on the Internet [38,44], the gauge installed in the marina and GNSS measurements (Figure 3) were used for determination of the safety contour, which is a boundary of bathymetric surveys in coastal areas.

### 2.4. USV Used for Bathymetric Surveys in Very Shallow Water

**Figure 4:**
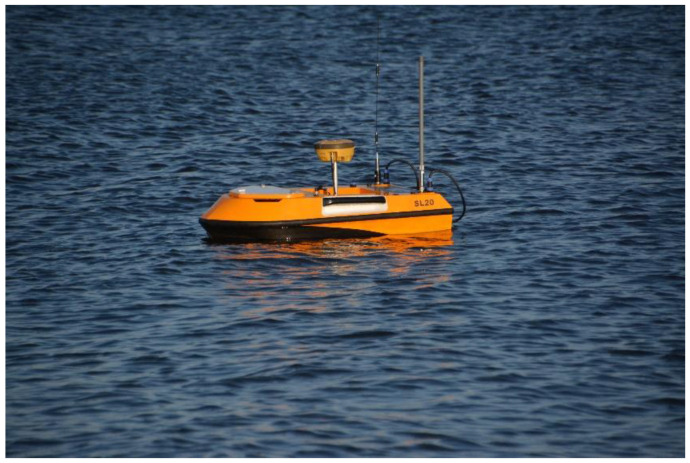
USV OceanAlfa SL20 during bathymetric survey.

During the surveys in shallow water areas, where USVs of draught of a dozen cm or so are used, the security isobaths should be determined where the USV security contour will be one of them—at the current water level. USV OceanAlfa SL20 is presented in Figure 4—it performed the bathymetric measurements in the water area adjacent to the municipal beach in Gdynia.

Table 1 presents the basic USV parameters in respect to its route along the sounding lines in shallow water.

Due to the bathymetric surveys planned on a very shallow basin and carried out by a USV, the safety contour was established within the range of 0–0.5 m with a resolution of 5 cm. Its selection is dependent on the size of the USV.

## 3. Results and Discussion

### 3.1. Area of the Research

**Figure 5:**
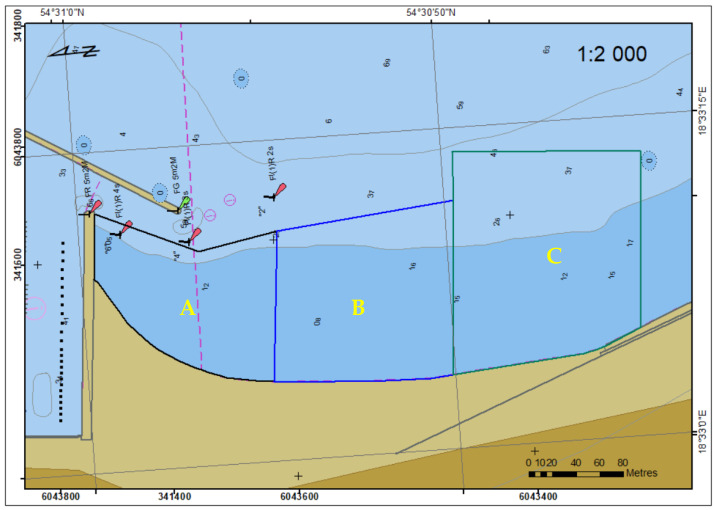
Sounding area divided into three parts.

The 450 m long beach was divided into three parts and each of them was surveyed on one day. The survey lines were planned in a parallel arrangement, perpendicular to the contour lines and the coastline, at a distance of 5 m. Their length varied: 75 m in the northern part, close to the fairway to the marina, 100 m in the middle part and 150 m in the southern part, the farthest from the marina (Figure 5).

The measurements in the middle part (B) were performed on the first sounding day. The profiles were planned on the basis of the coastline course in the ENC, keeping a 10 m margin—the distance from the coast. Due to the high water level, the profile distance from the temporary coastline was larger. Therefore, it was decided that the surveyed area border would be established by reconnaissance on the second and the third day (the southern (C) and the northern (A) part, respectively). The USV was guided in manual mode from the beach with visual supervision to a depth enabling safe navigation and survey.

**Figure 6:**
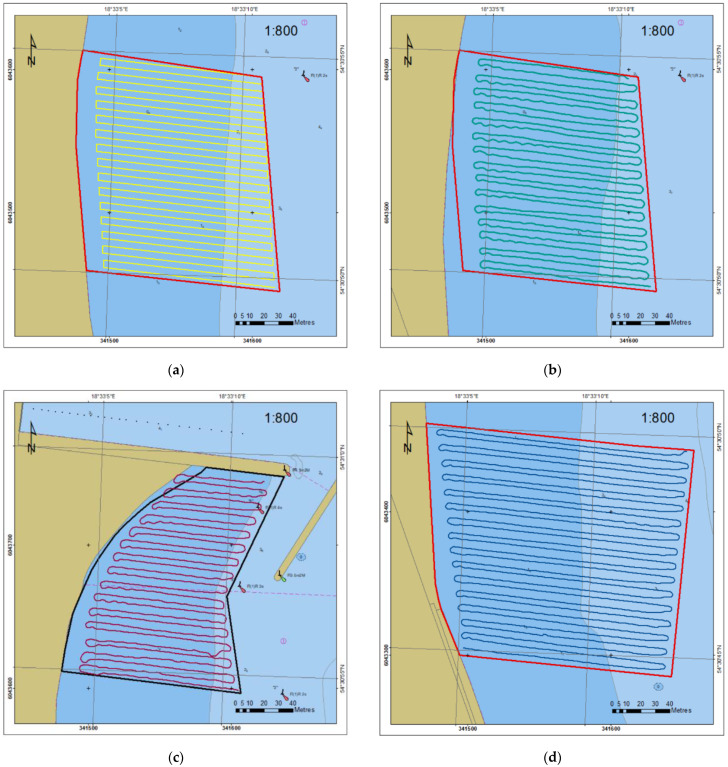
Planned lines in area B (**a**) and the trajectory of the USV in restricted areas: B (**b**), A (**c**) and C (**d**).

Figure 6 presents the trajectory of the USV during bathymetric surveys. The red line limits the area B on the basis on the ENC PL5GDYNA ed. 2018 (a) and ed. 2020 (b). Figure 6c,d present the trajectory in areas A and C, respectively.

### 3.2. Determination the Sounding Boundary

DSBM was prepared on the basis of the ENC PL5 GDYNA and contour lines were determined in the range of 0–0.5 m with a 5 cm interval. Depths in Mean Sea Level (MSL) vertical datum was related to the tide-gauge located in Kronstadt, according to the National Spatial Reference System [33]. Next, the current water level available from water level station located in Gdynia marina was located online [38,44].

Throughout the four-day period, major fluctuations of the water level in the range of 513–535 cm in local vertical datum were observed. Table 2 presents the water level in the morning, when bathymetric soundings were realised.

For determination of the depth in bathymetric surveys, the actual water level can be referred to the measured depths of the so-called chart datum, which for the PL-KRON86-NH height system, amounted to 508 cm. To do so, the following formula should be used [45,46,47,48]:

d’ = −(d + Δd_ED_ ± Δd_CD_) (1)

where d’ is normal height of the point measured by the echo sounder in the PL-KRON86-NH height system (cm), d is depth measured by the echosounder (cm), Δd_ET_ is draft of the echo sounder transducer (cm), Δd_CD_ is a depth correction referred to in the chart datum in the PL-KRON86-NH height system (cm), which needs to be added where the averaged sea level (d_SL_) does not exceed 508 cm; otherwise, it needs to be subtracted. The correction Δd_CD_, which is determined based on the following equation, requires additional explanation:

Δd_CD_ = 508 cm − d_SL_ (2)

where d_SL_ is averaged sea level observed on a tide gauge between consecutive full hours in the PL-KRON86-NH height system (cm).

To determine the current safety contour, the corrected depth should be reduced to the current sea level. When water is high, the safety contour will move towards the coast. When water is low, the sounding area boundaries recede from the coast. At the sea level higher than the security isobaths, the bathymetric soundings will be realised in the area marked in the ENC as a land area. Therefore, it is necessary to choose arbitrarily one of the two solutions: either to reduce the sounding area to the coastline on the basis of ENC or to make reconnaissance.

For determination of the safe contour, on the basis of the water level in the beginning the working (sounding) day, depth corrections should be taken into consideration as presented in Table 3. The depth clearance (20 cm) limits the contour line determining the sounding boundary in coastal area.

In high water levels, the sounding area can be limited up to the coastline or by bathymetric reconnaissance. In low water levels, the boundary will be further from the coastline. Please note that the determined contour line is the shallower point that the USV can take place. It can be a bow or a side. The area of sounding has to be additionally limited by the distance between a GNSS receiver antenna and a bow, taking into consideration USV manoeuvering parameters.

**Figure 7:**
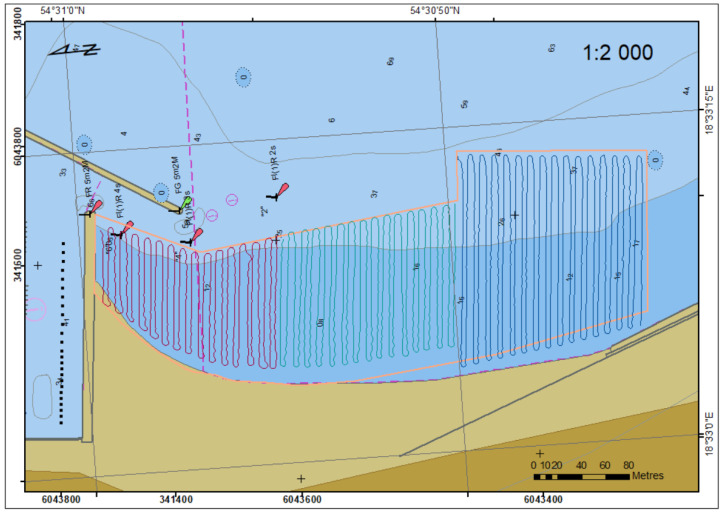
Sounding region with the USV’s trajectory.

Complete trajectory of the USV is shown in Figure 7.

**Figure 8:**
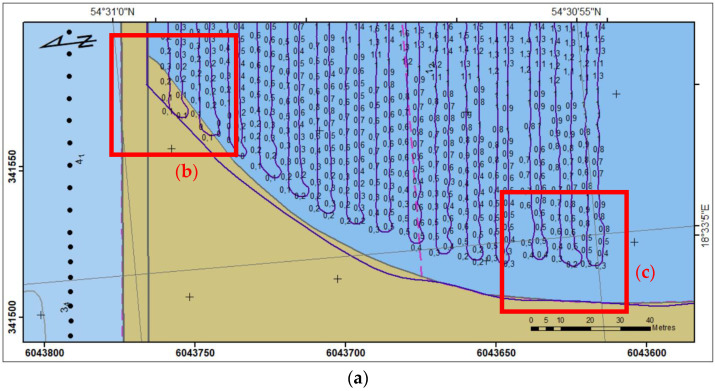

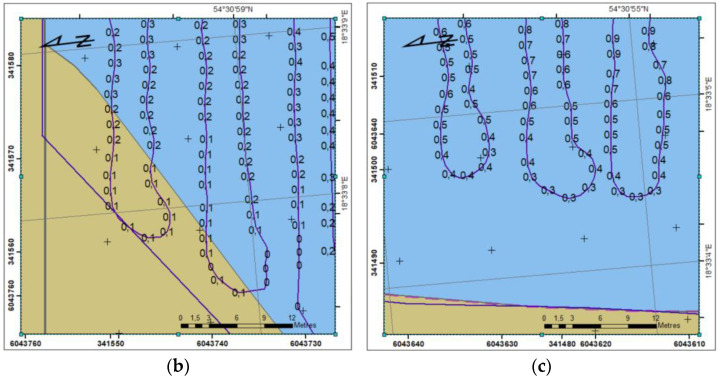
Sounding region A (**a**) with the USV’s trajectory; selected parts (**b**) and (**c**).

Below (Figure 8) shows sounded region A with USV trajectory, and measured depth. Although the trajectory is on land area (b), in current water level, coastal changes and bathymetric reconnaissance, soundings were as close to the coastline as it was possible. Taking into consideration seabed fluctuations, at the end of the area (**c**) distance to the coast is 12 m.

**Figure 9:**
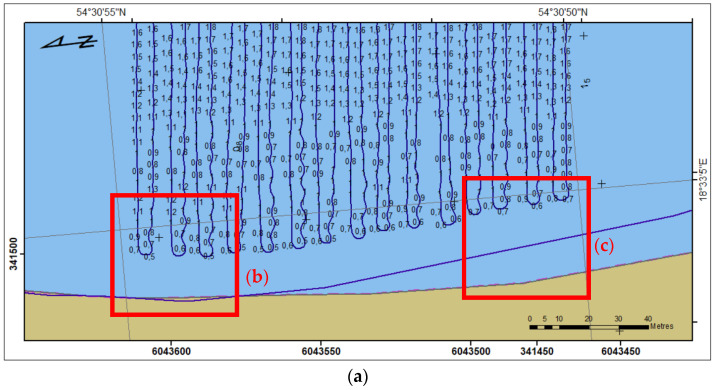

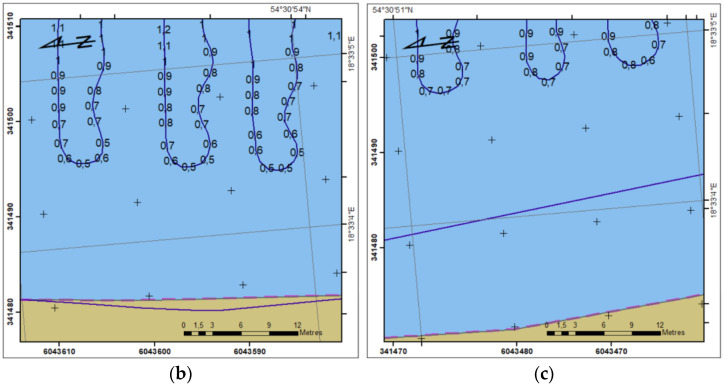
Sounding region B (**a**) with the USV’s trajectory; selected parts (**b**) and (**c**).

**Figure 10:**
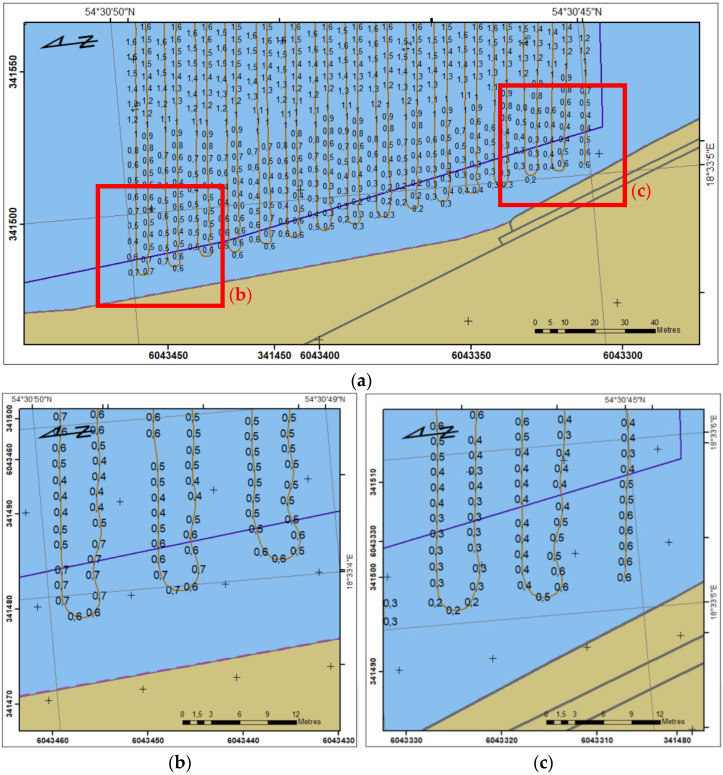
Sounding region C (**a**) with the USV’s trajectory; selected parts (**b**) and (**c**).

Figure 9 and Figure 10 present sounding regions B and c, respectively, with land area based on ENC edited in 2018 and sounding area limited by coastline based on ENC edited in 2021. The distance between the sounding area and coastline is a result of shallows (Figure 9) and stones (Figure 10) on the bottom.

## 4. Conclusions

Planning bathymetric surveys consists of several factors, e.g., economic, logistical and hydrometeorological. The method of measurement performance is determined by the availability of technical measures, which include a vessel and an echosounder. A USV equipped with an SBES makes it possible to perform measurements in an inshore area up to a very small depth, while ensuring the vessel’s navigational safety. The measurement is quick, especially a measurement performed in automatic navigation mode.

It was assumed that the survey profiles in the inshore area would be planned with the use of the ENC; therefore, such a cell must be developed for the area. The method presented in the paper is useful in a region where regular bathymetric measurements required for the basin are performed. Regular measurements are also performed in basins where the seafloor relief is varied, e.g., in ones with a tombolo. Measurements are usually performed twice: before the dredging work, to determine the volume of the bottoms for dredging, and after the work completion.

The use of a USV, guided along the planned profiles in the automatic navigation mode, enables the quick performance of the survey. One should note the coastline diversity, which results from geomorphological changes and the water level. With access to hydrological data, especially the eco-hydrodynamic model, a water level forecast performing the measurements at a high water level will help to acquire geospatial data as close to the coastline as possible.

## Figures and Tables

**Table 1:** Technical specification of the OceanAlpha USV SL20.

Parameter	OceanAlpha USV SL20
Hull material	Carbon fiber
Dimension	105 cm × 55 cm × 35 cm
Draft	15 cm
Propulsion	water-jet propulsion
Survey speed	2–5 kn (1–2.5 m/s)
Max speed	10 kn (5 m/s)
Positioning (standard—not used)	u-blox LEA-6 series
Positioning (used in manoeuvering)	Topcon HiPerII
Heading	Honeywell HMC6343
Echosounder	Echologger series SBES

**Table 2:** Water level [cm] in Gdynia marina.

Time Area	26.05.2020 B	27.05.2020 -	28.05.2020 C	29.05.2020 A
8.00	517	514	530.5	528
9.00	518	512.5	531	531
10.00	519	513	534	531
11.00	519	512.5	535	531.5
12.00	519.5	514.5	535	532
13.00	520	515.5	535	533
14.00	520.5	516.5	535	535

**Table 3:** Water level and suggested depth corrections.

Time	26.05.2020	27.05.2020	28.05.2020	29.05.2020
Water level [cm]	519	513	535	531
Depth correction [cm]	−20	−15	−35	−30
Contour line [cm]	0	5	0	0

## Data Availability

Not applicable.
